# The passive avoidance task ameliorate the toxic effects of bisphenol A on dopamine D1 receptor density in hippocampus, amygdala, and cerebellum of male rats

**DOI:** 10.1002/brb3.2942

**Published:** 2023-03-06

**Authors:** Mahnaz Taherianfard*, Saiedeh Ahmadijokani

**Affiliations:** ^1^ Physiology Division of Basic Science Department, School of Veterinary Medicine Shiraz University Shiraz Iran

**Keywords:** bisphenol A, dopamine d1 receptor, immunohistochemistry, passive avoidance task, rat

## Abstract

**Introduction:**

Dopamine D1 receptor seems to play a role in mediating plasticity. Therefore, the present study aimed to investigate the effects of passive avoidance tasks postexposed to BPA on dopamine D1 receptor density in the hippocampus, amygdala, and cerebellum of male rats.

**Methods:**

Thirty‐five male Sprague–Dawley rats weighing 220.300 g, in standard light‐dark 12 h light/12 h dark were used in the present study; water and food were ad libitum. Animals were divided into six groups. Administration of BPA 5 and 50 mg/kg/day were gavaged for 15 days. Learning and memory assessment were done by a shuttle box after 15 days of BPA administration. The density of the dopamine D1 receptor was investigated using an immunohistochemistry (IH) procedure. For determining the color difference in IH sections, Image Analyzer software was used. The data were analyzed by one‐way ANOVA followed by Tukey's as a post hoc test.

**Results:**

The data showed that BPA in both doses could significantly increase the density of dopamine D1 receptors in the hippocampus, amygdala, and cerebellum of male rats; learning in rats postexposed to BPA improves dopamine D1 receptor density significantly in three brain structures.

**Discussion:**

According to the results, passive avoidance learning and memory can improve the density of dopamine D1 receptors in the hippocampus, amygdala, and cerebellum of male rats.

## Graphical abstract

Bisphenol A is a xenoestrogen that could be destroyed dopaminergic neurons. It has many applications as polycarbonate plastic and epoxy resin production. Our results represent that passive avoidance learning and memory can reduce the toxic effect of bisphenol A on dopamine D1 receptors density in some brain structures.

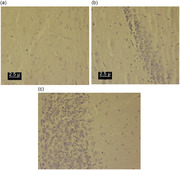

## 1 INTRODUCTION

Xenoestrogens are a group of synthetic chemicals that mimic the effects of the estrogen hormone. However, they are chemically distinct from the natural estrogen compounds produced by the endocrine glands. An example of these compounds is bisphenol A (BPA), which is used in polycarbonate plastics and epoxy resin production (Hall & Korach, 2002). BPA is composed of two phenolic groups that structurally are similar to beta‐estradiol and appear to have estrogen similarity (Li et al., 2015). A report has shown that prenatal exposure to low doses of BPA led to alteration of neuronal immigration and disruption of thalamocortical projections during cortical neural development (Itoh et al., 2012). Sadowski et al. (2014) reported that BPA in a higher dose leads to a diminish in the neuronal and glial cell number in vivo and in vitro. Wang et al. (2019) were reported that BPA could destroy dopaminergic neurons and lead to a decrease in dopamine synthesis in the brain. Another study pointed that BPA modulates their action through dysregulation of neurotransmitter systems sensitive to stress and endocrine disruption (Wiersielis et al., 2020). According to some studies, the brain neurogenesis level is affected by steroid hormones (Walters et al., 2010). The xenoestrogens through natural steroid receptors lead to disorders in the learning and memory system. Estradiol increases the number of synapses in the hippocampus, amygdala, and cerebellum prefrontal cortex (Weiss, 2007). The effects of steroid hormones in the brain are through estrogen and androgen receptors. In addition to gonadal steroids, neurosteroid synthesis was performed by neurons and glia in some brain regions that most significant of them are the amygdala, hippocampus, cerebellum, and prefrontal cortex (Diotel et al., 2018).

Dopamine is the dominant catecholamine neurotransmitter in the mammalian brain and controls many functions that modulate motor activity and intracellular ion transfers (Covey et al., 2017). Studies have shown that dopamine D1 receptors play a role in some aspects of behavior, including learning, memory, perception, and cognition (Messias et al., 2016). de Boer et al. (2019) data have shown that human dorsal striatal dopamine D1 receptors are involved in the modulation of instrumental learning biases.

The activity of the tyrosine hydroxylase enzyme in the hippocampal and amygdala regions reduces by BPA. It increases the activities of the central dopaminergic system (Brown, 2009). Therefore, BPA possibly changes the density of dopamine D1 receptors in the hippocampus, amygdala, cerebellum, and prefrontal cortex (Nagai et al., 2007). Therefore, the toxic effect of BPA in these brain regions was assessed in this study.

Many studies have been focused on the significant effects of BPA on structural and neurochemical changes of the brain (Inadera, 2015). On the other hand, many studies have shown that the dopamine D1 receptor could affect neural plasticity. The study showed there is a distinct dopaminergic pattern for good and poor performers that is critical for the expression of avoidance behavior. Moreover, it could be possible to switch from good to poor performers, and vice versa, by intra‐amygdala (BLA) injections of D1 receptor antagonist drugs in good performers or D2 receptor agonist drugs in poor performers (Antunes et al., 2020).

There are a few studies on the effects of learning and memory on the dopamine D1 receptor density. Thus, the present study aimed to examine the modulation effect of passive avoidance task on dopamine D1 receptors density in the hippocampus cornu ammonis 1 (CA1) region, amygdala, and Purkinje cell of the cerebellum in male rats preexposed to BPA.

## 2 MATERIAL AND METHODS

### 2.1 Animals and experimental design

Thirty adult male Sprague–Dawley, weighing 200–220 g, were used in this study. Food and water were available ad libitum during the study. The rats were housed under a 12‐h light/dark (lights on at six am and off at six pm) and controlled temperature (20 ± 4°C) condition. Animals were randomly allocated to six equal groups (*n* = 7). (1) NL‐ Sham (which received BPA vehicle without learning); (2) L‐Sham (which received BPA vehicle + learning); (3 and 4) NL‐BPA (which received BPA in two dose, without learning); (5 and 6) L‐BPA (which received BPA in two dose + learning). After BPA or BPA vehicle treatment, the passive avoidance procedure was began. The BPA 5, 50 mg/kg/day was used orally for 15 days (Hass et al., 2016). BPA was purchased from Sigma‐Aldrich Co.

### 2.2 Learning protocol

On the first day, all animals were individually subjected to 2 min of adaptation to the shuttle box. On the second and third days, the rats were placed in the light compartment box and 1 s after entering the dark compartment received a 0.6 mA foot shock for 1 s. On the fourth and fifth days, the procedure was similar as the second day except for foot shock. If the rats after 120 s did not move to the dark compartment during the third, fourth, and fifth sessions of experiments, they were considered as learned (Taherianfard et al., 2013). Passive avoidance learning and memory was according to RRID: RGD_1300432.

### 2.3 Sample preparation

In NL groups 24 after the last BPA exposure and in L groups twenty‐four hours after the end of passive avoidance learning sessions, all rats in the present study were anesthetized by sodium thiopental. The hearts were perfused with phosphate‐buffered saline (PBS) to flush blood out of the brain vasculature, then perfused with 4% formaldehyde. Then, the whole brain was removed and washed with normal saline. Brain fixed for 24 h in 10% formaldehyde in 0.1 M phosphate buffer (pH 7.4). It was followed by post fixing in 4% formaldehyde in 0.1 M phosphate buffer (pH 7.4). Tissue processing was accomplished in an automated tissue processor, and paraffin blocks were prepared. Furthermore, 5 μm serial sections of the amygdala, hippocampus CA1 region, and cerebellum Purkinje cell were made and mounted on 25% L‐lysine coated glass slides.

### 2.4 Immunohistochemical study

The immunohistochemical study was according to reference (Taherianfard et al., 2012). The difference was that primary antibodies against the dopamine D1 receptor (with a dilution of 1 × 10^−3^) were used (Abcam, UK). Our immunohistochemistry protocol was according to “**RRID: SCR_002396.”**

**FIGURE 1:**
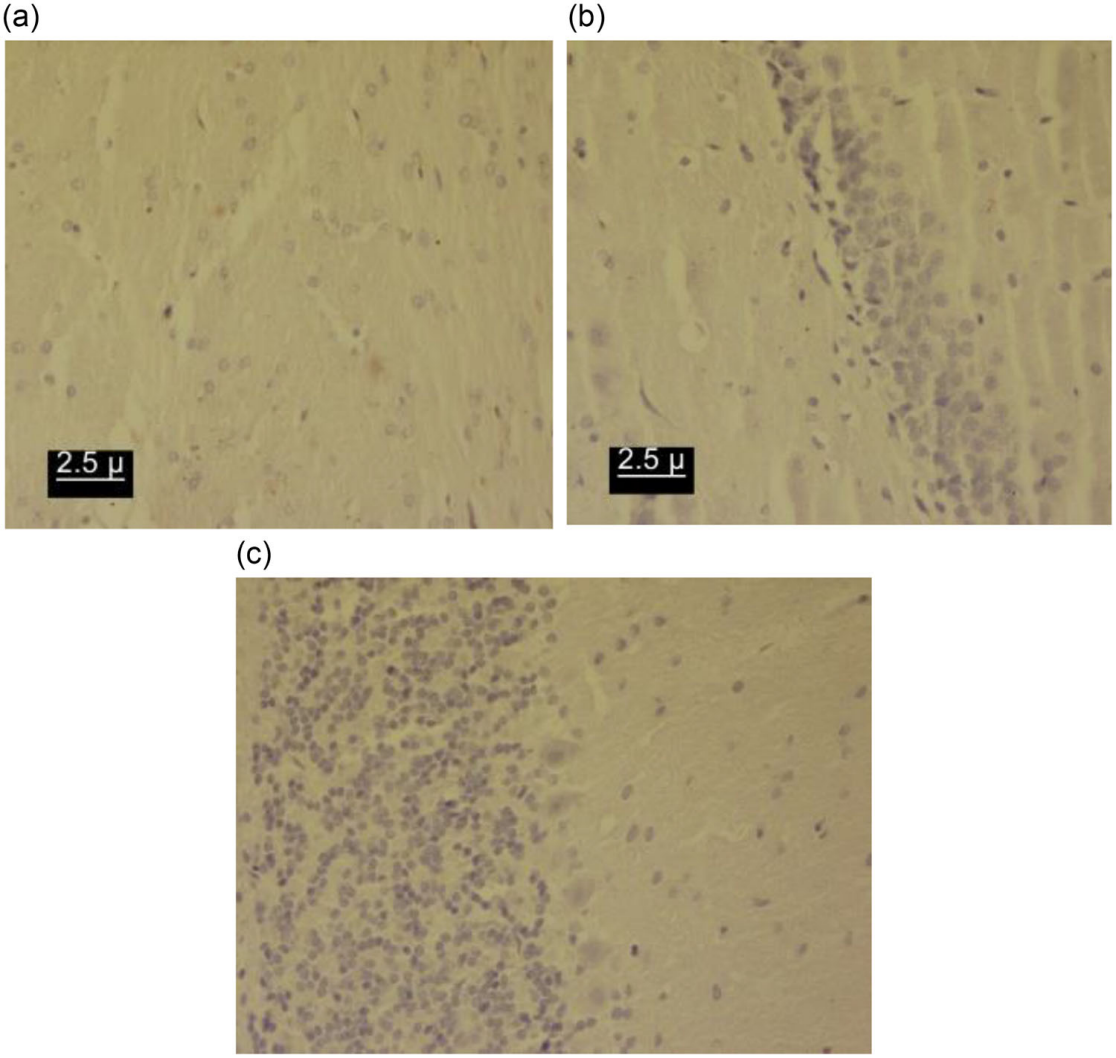
Photomicrograph of the negative control of the brain structure: (A) amygdala; (B) hippocampusCA1 region; (C) Purkinje cell layer of the cerebellum.

Negative control sections were incubated with PBS in the absence of primary antibodies, and immunoreactivity was not detected in the Purkinje cell layer of the cerebellum, amygdala nucleus, and CA1 region of the hippocampus (Figure 1). After preparing digital images, dopamine D1 receptor density was measured using the Image Analyzer (version 1.33) software (Rittscher et al., 2008). This program determines the density of D1 receptors according to three factors including hue, saturation, and intensity, and shows a number that has reverse relation with the receptor density. In other words, a lower number in the program represents higher receptor density and vice versa.

### 2.5 Statistical analysis

Statistical analysis was performed using the SPSS (version 21) software. Factorial ANOVA and One‐way ANOVA were used, and Tukey's as the post hoc test was performed to determine the difference between groups. Significant level was *p* < .05, and data were reported as mean ± standard error of the mean (SEM).

All the procedures involving animal subjects were reviewed and approved by the Institutional Research Ethics Committee of the School of Veterinary Medicine of Shiraz University (code# 94GCU2M1755).

## 3 RESULTS

**FIGURE 2:**
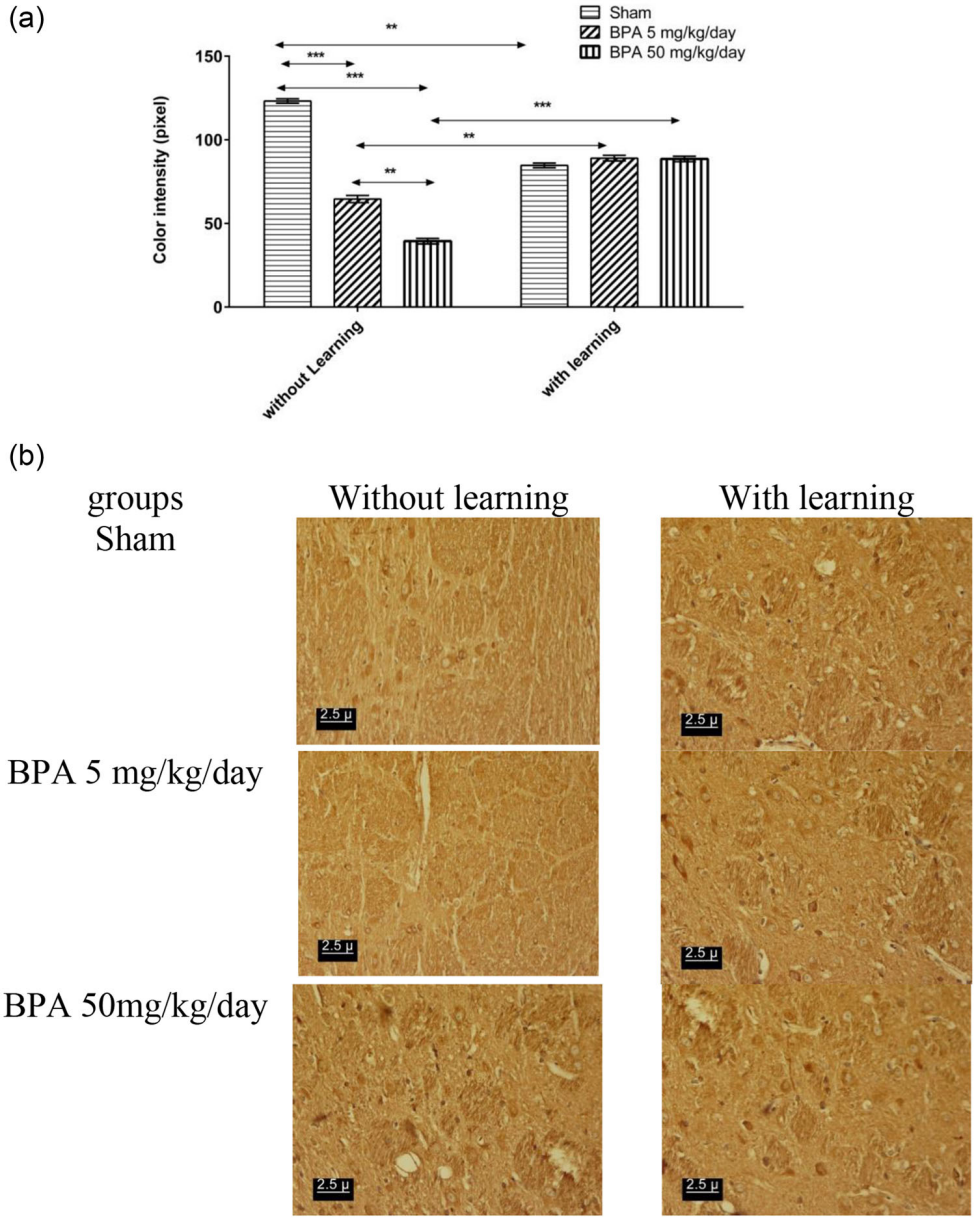
(a) Color intensity of immunohistochemistry staining of amygdala slides of rats in different groups which inversely demonstrates dopamine D_1_ receptor density (low number represent the high density of receptor and vice versa). (b) Photomicrograph of effect of learning and memory on density of dopamine D_1_ receptor pre exposure to BPA in amygdala of male rat in different groups. ** *p* < .01 significant level; *** *p* < .001 significant level. Data were showed as Mean ± SEM

**FIGURE 3:**
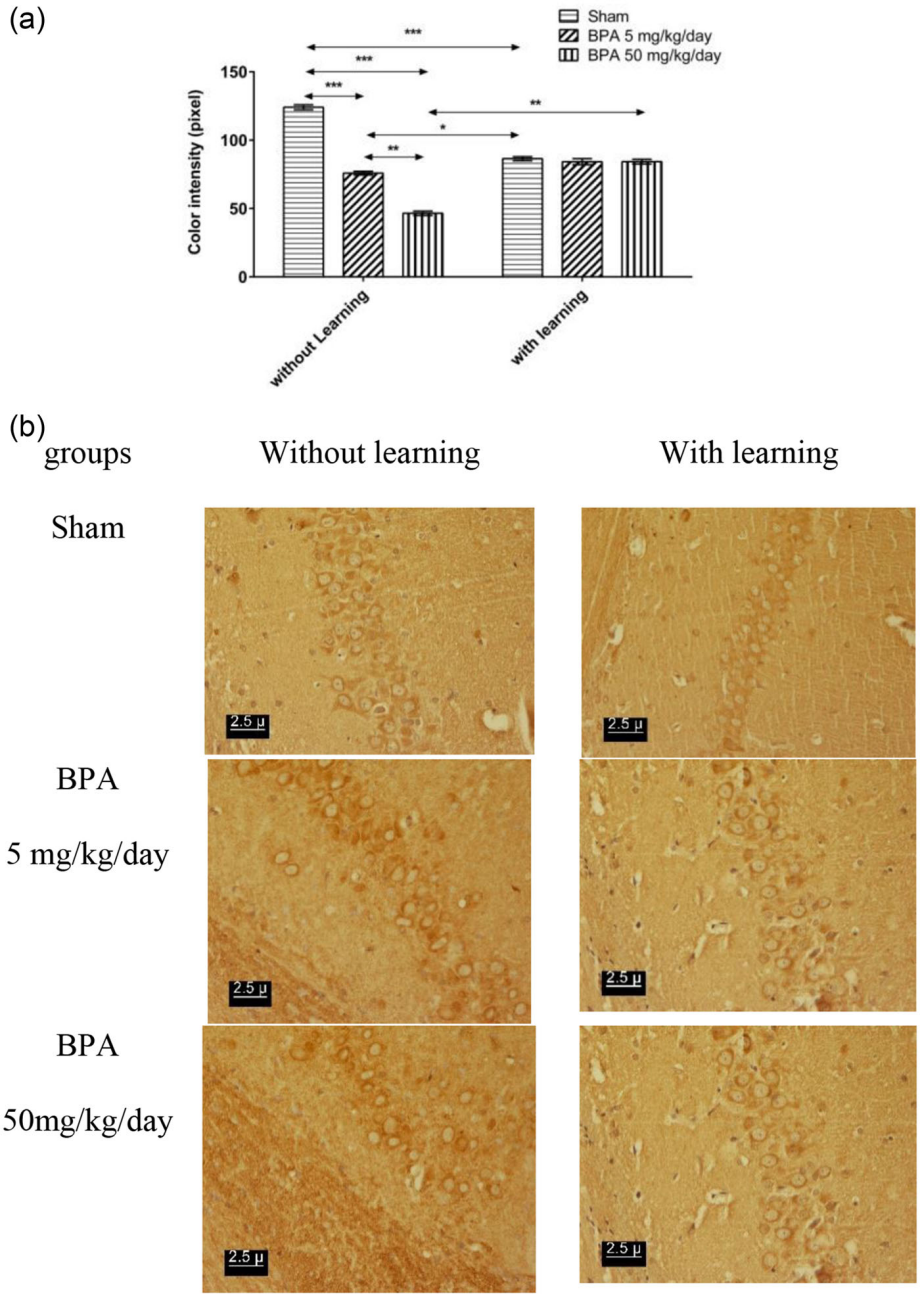
(a) Color intensity of immunohistochemistry staining of hippocampal CA1 area slides of rats in different groups which inversely demonstrates dopamine D_1_ receptor density (low number represent the high density of receptor and vice versa). (b) Photomicrograph of Effect of learning and memory on density of dopamine D_1_ receptor pre exposure to BPA in hippocampal CA1 area of male rat in different groups. * *p* < .05 significant level; ** *p* < .01 significant level; *** *p* < .001 significant level. Data were showed as Mean ± SEM

**FIGURE 4:**
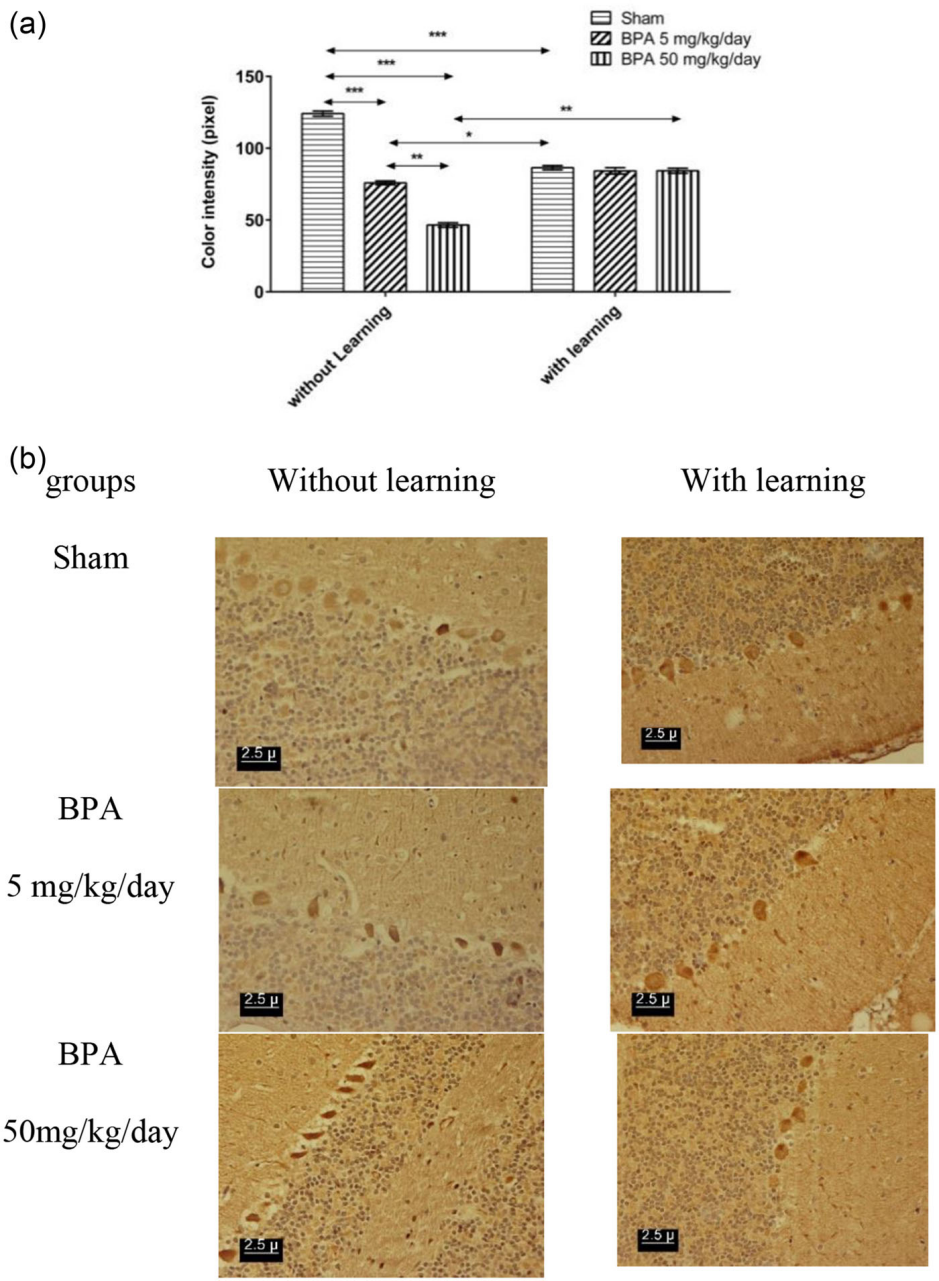
(a) Color intensity of immunohistochemistry staining of cerebellum Purkinje cell slides of rats in different groups which inversely demonstrates dopamine D_1_ receptor density (low number represent the high density of receptor and vice versa). (b) Photomicrograph of Effect of learning and memory on density of dopamine D_1_ receptor pre exposure to BPA in cerebellum Purkinje cell of male rat in different groups. * *p* < .05 significant level; ** *p* < .01 significant level; *** *p* < .001 significant level. Data were showed as Mean ± SEM

Administration of BPA in both doses showed a significant increase (*p* < .001) in the density of dopamine D1 receptors in the amygdala, hippocampal CA1 region, Purkinje cell of the cerebellum in comparison to the NL‐sham, while the BPA 50 mg/kg/day significantly increased (*p* < .01) the density of dopamine D1 receptor relative to the BPA 5 mg/kg/day in the amygdala, hippocampal CA1 region, Purkinje cell of the cerebellum (Figures 2, 3, and 4a). Passive avoidance learning did not lead to a significant difference in the density of dopamine D1 receptors in both L‐BPA groups compared to L‐sham in the amygdala, hippocampal CA1 region, Purkinje cell of the cerebellum. Moreover, in L‐sham the density of dopamine D1 receptor significantly (*p* < .01) was higher than NL‐sham in the amygdala, hippocampal CA1 region, Purkinje cell of the cerebellum. But the density of dopamine D1 receptor significantly (*p* < .01) was lower in both L‐BPA than both NL‐BPA in the amygdala, hippocampal CA1 region, Purkinje cell of the cerebellum (Figures 2, 3, and 4a). Figures 2, 3, and 4b represent the photomicrograph of the brain sections in all groups in the amygdala, hippocampal CA1 region, Purkinje cell of the cerebellum; the darker brown stain represents the high density of the receptor.

## 4 DISCUSSION

In a previous study, we found that administration of the BPA at 5 and 50 mg/kg/day for 15, impaired learning and memory in passive avoidance learning and memory (Taherianfard et al., 2013). In rodents, learning behavior disorders have been reported after exposure to BPA in both sexes. The results have shown that BPA can intervene in the formation of synaptic spines in the hippocampus and prefrontal cortex, which has clinical implications (Carr et al., 2003). The findings of Xu et al.’s (2010) showed that prenatal exposure to BPA could significantly weaken spatial memory and passive avoidance memory during the postprandial developmental stages and adolescence in male children (Xu et al., 2010). Poimenova et al. (2010) reported that long‐term exposure to a safe BPA dose could weaken spatial diagnostic memory for Y‐maze in middle‐aged rats in both genders (Poimenova et al., 2010). In male rats, BPA alleviates the passive avoidance memory and reduced choline acetyltransferase in the hippocampus (Miyagawa et al., 2007).

According to the present study, BPA can cause an increase in the density of dopamine D1 receptors in the hippocampus CA1 region, amygdala nucleus, and Purkinje cell of the cerebellum. Tando et al. (2007) showed that the administration of BPA low dose before and after birth reduces the tyrosine hydroxylase enzyme (key enzyme for dopamine synthesis) in the substantia nigra (Tando et al., 2007). Ishido et al. (2004) described that intracisternal injection of BPA 0.2 to 20 μg/kg/day, for 5 days, affected the activity of the central dopaminergic system of rats (Ishido et al., 2004). Disorders induced by BPA in animals; depend on the dose and the period of exposure (Rice & Barone, 2000). Chronic exposure to BPA induces the activation of G protein by dopamine; it was observed that the blockade of the dopamine D1 receptor revealed a reverse effect on the activation process of G protein. These findings indicate the mediation of the activity of D1 receptor dopamine following pre‐ and postbirth exposure in signal transduction (Suzuki et al., 2003). In addition, prolonged exposure to BPA can increase mesolimbic dopamine, resulted in the synaptic plasticity, and impair the development of the nervous system (Dessi‐Fulgheri et al., 2002). Obata (2006) stated that BPA increased hydroxyl radical, which, in turn, increased dopamine‐releasing in the brain striatum of male Wister rats. Imidaprilat as Angiotensin‐converting enzyme inhibitors; can protect the nervous system by reducing the production of hydroxyl radical‐induced by BPA, leading to a decrease in dopamine efflux (Obata, 2006).

Prenatal exposure to BPA at doses of 4 and 40 mg/kg/day affects the brain's neurotransmitter (Honma et al., 2006). Miyatake et al. (2006) reported that in vivo exposure of mouse glial cells and astrocytes to BPA at a low dose increased dopamine secretion (Miyatake et al., 2006).

Narita et al. (2007) observed that exposure to BPA in different life periods of mice had various effects. They found that BPA had the most adverse effects in the embryonic and infantile periods of the animals’ life. They added that exposure to BPA could cause the potentiation of the dopaminergic system (Narita et al., 2007). According to the current study, it seems that BPA in both doses leads to an increase in dopamine D1 receptors density. Therefore it may increase the expression of the receptor in the brain structures or upregulate the receptors.

In the present study, passive avoidance learning in rats postexposed to BPA improve the density of dopamine D1 receptor in the amygdala nucleus, hippocampus CA1 region, and Purkinje cell of the cerebellum. It means that passive avoidance learning and memory can improve the toxicity of BPA. The other experiment provides evidence of a positive relationship between the rate of turnover/recovery of newly synthesized D1 dopamine receptors in the mPFC and animal's general cognitive abilities (Wass et al., 2018). The novelty of our finding is that the previous study only investigated the effects of dopamine agonists and antagonists on learning and memory. Nevertheless, our results have shown the effects of learning and memory on the dopaminergic system. So, we found that dopamine D1 receptors density can be affected by passive avoidance learning.

Fedotova and Sapronov (2012) investigated the role of the dopamine D1 receptor in learning and memory processes during the estrous cycle of adult female rats by passive avoidance learning. The results indicated that D1 receptor dopamine could mediate learning and memory processes during the sexual phase (Fedotova Iu & Sapronov, 2012). Evidence suggests that a low dose of BPA may interfere with the formation of synaptic plasticity at the back of the striatum in rats. Exposure to the low dose of BPA in 28‐day male rats led to disorder in the LTP formation. This defect in the formation of LTP and LTD can be avoided by changing the performance of dopamine receptors (Zhou et al., 2011). Oral exposure to BPA 100 and 500 μg/kg/day, 7 days before birth and 36 days after birth, reduced the attention in the Y‐maze test (Tian et al., 2010).

## 5 CONCLUSION

The BPA molecule has structural features that allow it to bind to 2 estrogen receptor (ER) subtypes (i.e., ERα and ERβ) despite that BPA displays 1000‐ to 2000‐fold less affinity to the ERs than does 17β‐estradiol (E2), the most active estrogen (Acconcia et al., 2015).

In the present study, BPA as an estrogen partial agonist led to dopamine D1 receptor up‐regulation. More detailed investigations are suggested on the dopamine D1 receptor gene expression. In the present study it was also shown that learning could improve the toxic effects of BPA. This could be attributed to the increase in the brain estrogen production caused by learning and memory processes.

## AUTHOR CONTRIBUTIONS

Mahnaz Taherianfard: study design, conceptualization, writing‐original draft, writing‐review, data analysis, funding acquisition and editing. Saiedeh Ahmadijokani: methodology and data acquisition.

## CONFLICT OF INTEREST STATEMENT

The authors declare no conflicts of interest.

### ETHICS STATEMENT

The animal handling was conducted according to the Ethical Committee for Animal Experiments at Shiraz University.

**Animal welfare code**: 94GCU2M1755.

### PEER REVIEW

The peer review history for this article is available at https://publons.com/publon/10.1002/brb3.2942.

## Data Availability

The data that support the findings of this study are available from the corresponding author upon reasonable request.
